# The association between caries related treatment needs and socio-demographic variables among young Israeli adults: a record based cross sectional study

**DOI:** 10.1186/s13584-018-0222-3

**Published:** 2018-05-09

**Authors:** Dan Henry Levy, Alon Livny, Harold Sgan-Cohen, Nirit Yavnai

**Affiliations:** 1 Medical Corps, Israel Defense Forces, Ramat Gan, Israel; 2 The Shraga Segal Dept. of Microbiology, Immunology and Genetics, Ben Gurion University of the Negev, Beer Sheva, Israel; 3 Department of Community Dentistry, Hebrew University-Hadassah School of Dental Medicine, Jerusalem, Israel

## Abstract

**Backgroud:**

The need for dental treatments, especially those related to dental caries, may be associated with and influenced by a wide range of demographic variables. The aim of this study was to describe caries related treatment needs among young Israeli adults and the association with several socio-demographic factors, including socio-economic cluster (SEC), intellectual capabilities, ethnicity and other variables.

**Methods:**

Data were collected from dental records of army recruits between 2012 and 2013. We cross-examined data regarding dental treatment needs with socio-demographic variables: age, gender, SEC, intellectual capability score (ICS), birth place of participant and parents, education and immigration.

**Results:**

Data received regarding 13,398 combat recruits during their first four months of military training. Most subjects were males (92.4%), with a mean age of 18.9 years. 10.8% were immigrants, with 12.2 years living in Israel before their recruitment. Only 17.7% had no dental treatment needs. Mean number of teeth needing treatment was: for restorations 1.96 ± 2.59, for root canal therapies 0.07 ± 0.44 and for extractions 0.05 ± 0.28. Low ICS scores and low SEC group were significantly associated with higher treatment needs (*P* < 0.001). Statistically significant higher treatment needs were observed among participants who originated from immigrant families. In a multivariate analysis (Generalized Linear Model), gender, age, ICS score, SEC group and country of birth were found as independent predictors for number of restorations needed.

**Conclusion:**

Socio-demographic variables significantly influence dental treatment needs and should be taken into account when preparing intervention programs in this population.

## Background

The understanding and level of global oral health has dramatically improved over the past 30–40 years. Nevertheless alarming disparities remain between and within countries [1]. The epidemiology of dental diseases has clearly been described as a web of connecting factors, including biological, social, psychological, economic, environmental and other variables [2]. An abundance of the literature has emphasized the role of Socio-Economic Cluster (SEC) on oral health [3, 4]. SEC has been described as those social determinants (including education, economic, social and others), which influence health. A predominant factor, almost always included as an SEC marker is *education*. This has been extensively studied in a wide spectrum and diversity of cultures, including Japan, Finland, Iran and more [5–7]. Commonly, education is related to intelligence, albeit not always. To date, locating studies associating intelligence with oral health has been largely futile [8].

The military population in Israel is large and standard archived measures include an estimation of intelligence. Hence we believed that this might provide a unique opportunity to investigate the relationship between intelligence and oral health.

A previous study from Israel assessed caries levels among 581 young adults during their military service. Among other findings, it showed that caries experience was higher among immigrants from the former USSR, compared to native Israelis, which were also more likely to attend regular dental appointments throughout their lives [9]. An additional study of young Israeli adults showed higher levels of caries experience among soldiers of Ethiopian or former USSR origin, and among subjects with less than 12 years of schooling [10].

The military population in Israel is large, and serve as a reliable source of data for demographic and epidemiologic studies among young adults, partially because military service in Israel is compulsory for both young men and women. Standard archived measures include an estimation of intelligence, which is derived from a validated indicator – the *Intellectual Capability Score (ICS)*. This indicator serves as a sorting factor for different military roles. We believed that having this data might provide a unique opportunity to investigate the relationship between intelligence and oral health.

## Methods

This is a records based cross sectional study among combat soldiers recruited between 2012 and 2013. Data were drawn from records of mandatory dental examinations which took place during the first 4 months post recruitment, in training bases. Original data were collected by approximately 20 military dentists, and included a detailed treatment plan, from which we have extrapolated the main dependent variables: the number of teeth in need of dental restorations, root canal treatments (RCTs) or extractions due to caries (excluding third molars). We utilized these variables as a proxy for oral health.

All examinees were healthy (a basic requirement for combat soldiers). The treatment plan was made after an examination performed in the bases, according to the guidelines of the dental department in the IDF medical corps, and included two bitewing radiographs for all examinees. Periapical radiographs were taken in cases of deep caries or former endodontic treatment. Quality control of these treatment plans and adherence to these guidelines were checked seasonally by the regional military chief dental surgeons.

Demographic independent variables were derived from IDF’s central demographic database and included: age, gender, birth country of participant and of both parents, years since immigration, and education level. SEC was determined according to place of residence, using the Israel Bureau of Statistics ranking of 1–10 (10 being the highest most well off score) [11]. For ease of presentation and analyses, this variable was categorized into low (1–4), medium (5–7) and high (8–10) SEC levels.

Intellectual capability in the IDF is assessed by ICS (also known as Initial psycho-technical evaluation/'Dapar’). This measure, established during a pre-draft screening, is ranked on a 9-point scale (between 10 and 90, with 10-point increments).

The validity of the ICS as a measure of general intelligence has been previously demonstrated to exhibit a correlation > 0.8 with the Wechsler Adult Intelligence Scale total IQ [12]. ICS is comprised of 4 time-limited tests with progressing difficulty. The tests are (1) a modified Otis-type verbal intelligence test, appraising ability to understand and execute verbal instructions; (2) verbal analogies, a modified version of the “similarities” subtest of the Wechsler Intelligence Scales, measuring verbal abstraction and categorization; (3) mathematical knowledge, assessing mathematical reasoning, concentration, and concept manipulation; and (4) nonverbal analogies, a modified version of Raven’s Progressive Matrices that evaluates nonverbal abstract reasoning and visual–spatial problem-solving abilities [13, 14].

For univariate analyses purposes, we dichotomized the scores according to the 75-percentile cut-off into low (scores under 70) and high (70 or more).

Data were analyzed by statistical analyses software (SPSS, version 21) with significance level set at α = 0.05. Univariate analyses included descriptive statistics (means, standard deviations and medians), Mann Whitney and Kruskal Wallis tests to evaluate associations between continuous non-normally distributed variables. Correlations between continuous variables was performed by using Pearson correlation coefficient. General Linear Model (GLM) multivariate analysis was applied to predict the restorative treatment needs. Independent variables that were significant in the univariate analyses were entered to the GLM.

## Results

The results shown are from files of 13,398 combat soldiers (92.4% male, mean age 18.92 ± 0.8). Median ICS score was 50 (range 10–90) and the median SEC score was 6.

Most of the examinees (11,957, 89.2%) were born in Israel; 4.6% (*n* = 611) have immigrated from the former USSR and 0.9% (*n* = 119) were born in Ethiopia. Immigrant participants had lived an average of 12.15 ± 5.6 years in Israel prior to recruitment (range 0.07–22.17). The majority of examinees (96.9%) had completed high school prior to their recruitment.

According to the examinations’ findings, 17.7% (2368) examinees were without need of any treatment. The mean number of teeth in need of restorations was 1.96 ± 2.59 per soldier (range 0–24), 0.07 ± 0.44 for RCTs (range 0–12) and 0.05 ± 0.28 for extractions (range 0–5). Association between socio-demographic variables and dental treatment needs are presented in Table 1. Higher mean number of needed dental restorations was found among males. However, gender was not significant in mean number of needed RCT’s or extractions. Significant associations were found between lower ICS and lower SEC groups and higher need for all three treatment categories.

**Table 1:** Associations between socio-demographic variables and dental treatment needs

		N	Num. of teeth in need of restorations		Number of teeth in need of RCTs		Number of teeth in need of extractions	
			Mean (SD)	*p* value	Mean (SD)	*p* value	Mean (SD)	*p* value
Gender	Male	12,375	1.99 (2.62)	< 0.001^a^	0.07 (0.45)	NS	0.05 (0.28)	NS
	Female	1023	1.63 (2.19)		0.06 (0.33)		0.04 (0.27)	
Country of birth	Israel	11,957	1.95 (±2.59)	< 0.001^b^	0.07 (±0.45)	< 0.001^b^	0.05 (±0.28)	< 0.001^b^
	Former USSR	611	2.52 (±2.74)		0.12 (±0.51)		0.08 (±0.35)	
	North America	316	1.22 (±1.91)		0.01 (±0.11)		0.01 (±0.13)	
	Ethiopia	119	3.13 (±3.34)		0.12 (±0.42)		0.08 (±0.32)	
	Other	395	1.64 (±2.36)		0.04 (±0.23)		0.04 (±0.23)	
ICS score	Low (< 70)	10,018	2.10 (2.69)	< 0.001^a^	0.09 (0.49)	< 0.001^a^	0.05 (0.31)	< 0.001^a^
	High (70–90)	3364	1.55 (2.24)		0.03 (0.22)		0.02 (0.19)	
SEC groups	Low (1–4)	3511	2.39 (2.97)	< 0.001^b^	0.12 (0.59)	< 0.001^b^	0.07 (0.35)	< 0.001^b^
	Mid (5–7)	5867	1.83 (2.43)		0.05 (0.33)		0.04 (0.25)	
	High (8–10)	2489	1.47 (2.05)		0.03 (0.23)		0.02 (0.20)	

*NS* not statistically significant

^a^Mann-Whitney test

^b^Kruskal-Wallis test

Post hoc tests revealed that subjects who were born in North America showed a significantly lower need for dental treatments compared to all other groups. Subjects who were born in Israel had significantly lower treatment needs compared to former USSR immigrants (in all three categories- *P* < 0.001), and compared to former Ethiopian immigrants (only for dental restorations and RCT’s, *P* < 0.001 and *P* = 0.03, respectively).

A multivariate Generalized Linear Model (GLM) revealed that male gender, older age, low ICS and low SEC, being born in former USSR countries or in Ethiopia, were all predictors for higher restorative need (*P* < 0.001, Table 2).

**Table 2:** GLM prediction model with number of teeth which needed dental restorations as the dependent variable

Independent variable		Relative risk	*P* value	95% Confidence Interval	
				Lower	Upper
Intercept		2.178	< 0.001	1.616	2.937
Age^a^		1.037	< 0.001	1.021	1.053
ICS^a^		0.991	< 0.001	0.991	0.992
SEC^a^		0.916	< 0.001	0.909	0.923
Gender	Male	1.141	< 0.001	1.083	1.204
	Female	1.000	–	–	–
Country of birth	former USSR^b^	1.074	< 0.001	1.035	1.114
	All other countries	1.000	–	–	–
Country of birth –	Ethiopia^b^	1.292	< 0.001	1.192	1.401
	All other countries	1.000	–	–	–

^a^Continuous variable

^b^Birth place of either the subjects or at least one of their parents

Similar analyses for RCT’s and extractions were not conducted due to the relatively low number of such treatments.

## Discussion

This study analyzed data regarding dental treatment needs and demographic variables of 13,398 young Israeli adults. Its predominant advantage is the large sample size which is optimally representative of Israeli young healthy adults. To the best of our knowledge, this is the first time this new database of computerized dental treatment records, which includes full and complete dental needs data, is being studied and published. Most of the soldiers in this sample were males (9:1 ratio), due to few females recruited to combat positions. We consistently demonstrated a reverse correlation between the level of SEC and ICS and higher dental treatment needs in all three treatment categories. The associations between socio economic status and dental needs have been established and described in previous studies [15–17], however, comprehensive studies regarding the correlation between dental caries and intellectual capabilities are few. Navit et al. tested 252 10–15 year old children for the correlation between IQ (according to Malin’s intelligence scale) and dental caries (DMFT index). No significant association between level of intelligence and caries prevalence was observed [18]. The current study offers a unique opportunity to explore a comprehensive database that enables a crossing of dental records with intellectual data. Our results found a statistically significant association between low intellectual capabilities and higher dental treatment needs. As there are almost no previous data regarding association between intelligence levels and caries prevalence, we may suggest that ICS serves here as an indirect marker of other, uncollected variables such as social or material deprivation, or educational level of the examinees’ parents. All these markers were already shown to be associated with caries prevalence [3–7, 19]; many studies suggest that children’s intelligence level is associated with parental education level (via genetic as well as environmental pathways) [20–22], and also with poverty [22–24]. However, the statistical significance we have shown could also be merely the result of the large sample size of our study (*n* = 13,398) rather than a difference so great that ICS could be useful for planning or evaluating interventions. Therefore, further studies should be performed, to study the association between ICS and caries prevalence, which is better measured directly by the DMFT (Decayed, Missing, and Filled Teeth) index, rather than by a proxy measure such as treatment needs.

Higher treatment needs were found among subjects whose parents came from Ethiopia or former USSR, indicating a clear need for preventive intervention among these two subgroups. These findings are similar to previous studies from Israel [10, 25–28]. Since both these populations were underrepresented in this study (for Ethiopian descendants: 1.7% among sampled subjects vs. 2.2% of the general Jewish population in 2013; for USSR origin: 12% vs. 15%) [11], this problem may be even more relevant in the general Israeli population. However, we could see that second generation subjects whose parents came from former USSR, had better oral health compared to subjects who immigrated themselves, suggesting a positive trend of improved health status among this subgroup, and possibly a marker of integration and assimilation.

The study has some limitations: we collected data regarding extractions but had no ability to ascertain whether the teeth were missing due to caries. If this had been available, it could have contributed to establishing a more thorough understanding of caries morbidity. Regarding the need for dental restorations, we could not exclude the need for restorations due to trauma or faulty restorations although we presume, judging by our experience that these are a minority that did not alter our results significantly.

We did not have access to data regarding oral hygiene, diet, smoking and other variables, which could shed light on causes for caries, and might further explain the relevance of ICS to untreated dental disease. Furthermore, all data collected were based on clinical and radiographic examination conducted by many military dentists with possible diversity in diagnosis of pathologies and their decisions regarding treatment needs. Examiners had received uniform training, but optimal calibration was not conducted. These examinations are mandatory but cases of refusal or missed examinations are possible and might have been undocumented.

At the present moment, dental health coverage in Israel is from birth to age 16 years. The intention is to eventually reach 18 years of age. Army recruits, therefore provide an optimal (albeit not maximal) age cohort for exploring wider dental health coverage. Results of the present study provide several indications for “at risk” criteria. These would include immigrants originating from Ethiopia and former USSR, lower SEC and low ICS groups.

The present study population has been utilized as representative for young adults in previous epidemiologic studies [24, 23, 10, 9]. Our results may therefore be considered as a baseline for future intervention programs. Dental caries is a chronic and progressive disease. Based on the recognition that Israeli military service is compulsory, and most immigrant soldiers live in Israel several years before induction, opportunities to prevent and control dental caries earlier in life exist and might lead to better oral health among recruits. Results of the present study indicate that interventions developed for the subgroups demonstrating higher needs, can serve as an important baseline against which to ascertain planning and optimal progress in the future.

The dental department of the IDF had focused in recent years on the enhanced treatment of two major populations- (1) Combat troopers; and (2) Low SES soldiers. For example, dental crowns and implants are provided to these soldiers while other mandatory service soldiers do not enjoy these benefits. Our study supports this rational by presenting evidence for the greater treatment needs in the Low SES population. This population also consists of (as expected) a high percentage of ‘lone soldiers’ (soldiers who immigrated without their parents) or immigrants with parents in Israel.

Furthermore, in the past, the dental department organized several intervention plans aimed at providing dental care for certain immigrant populations. We believe our results might support similar and enhanced plans in the future.

## Conclusion

Socio-demographic variables such as SEC, ICS and country of birth significantly influence dental treatment needs and should be taken into account when preparing intervention programs in this population.
